# Supplementary figures and images for: Characterisation of class VI TRIM RING domains: linking RING activity to C-terminal domain identity
Source: Life Sci Alliance. 2019 Apr 26;2(3):e201900295. doi: 10.26508/lsa.201900295 (PMC6487577; doi:10.26508/lsa.201900295)

Figure 2 Source data

2A

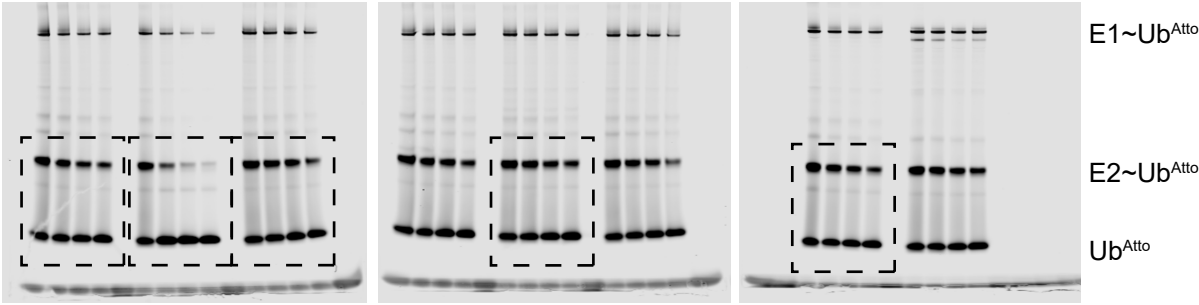

2C

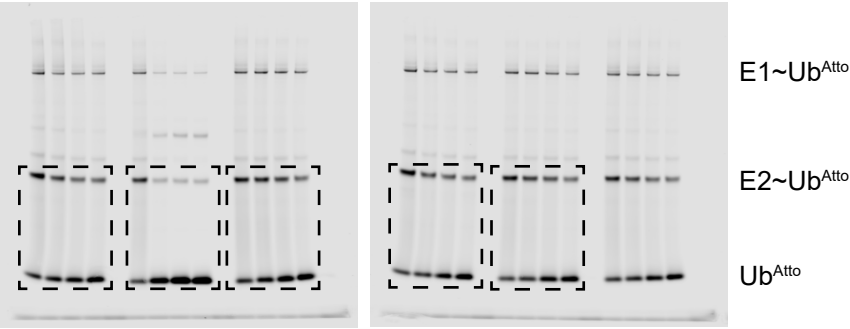

2E

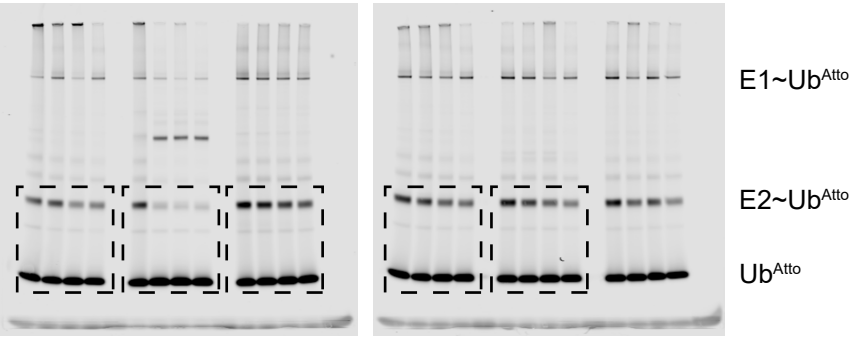

Supplement: Supplementary file 1 [file LSA-2019-00295_SdataF2.pdf]

Figure 3 Source data

3D

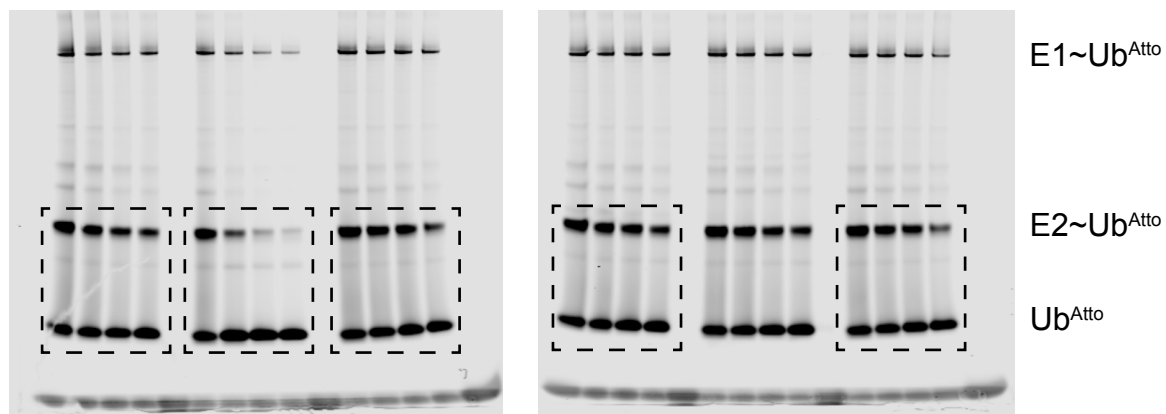

3F

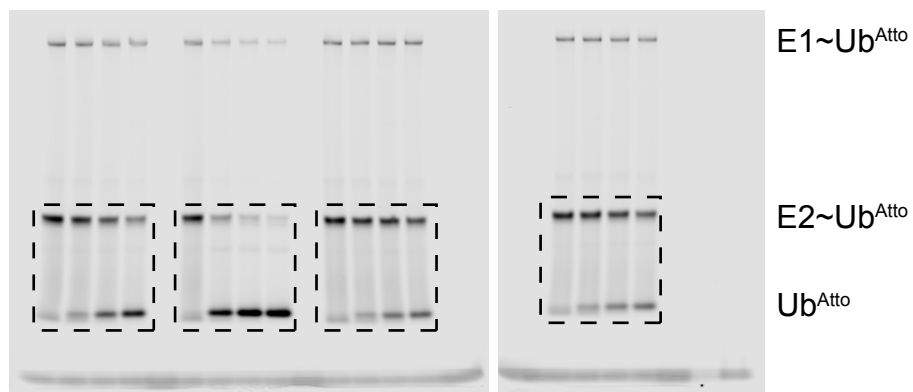

Supplement: Supplementary file 3 [file LSA-2019-00295_SdataF3.pdf]

Figure S2 Source data

S2A

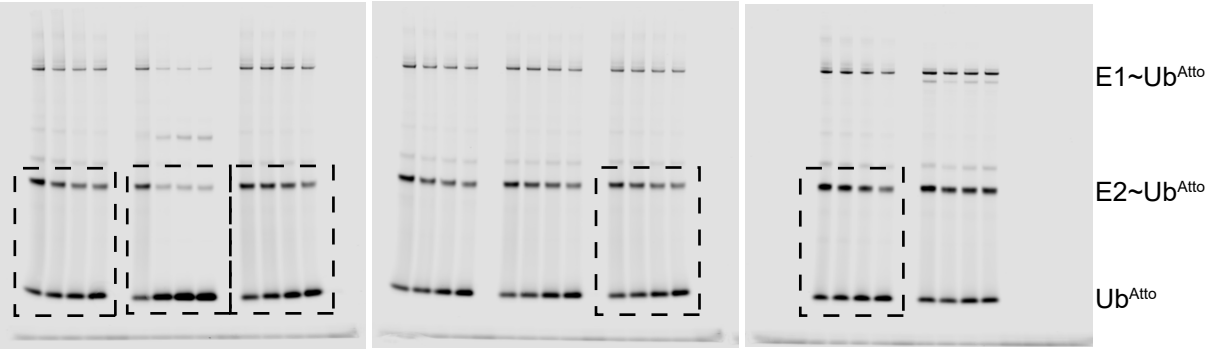

S2C

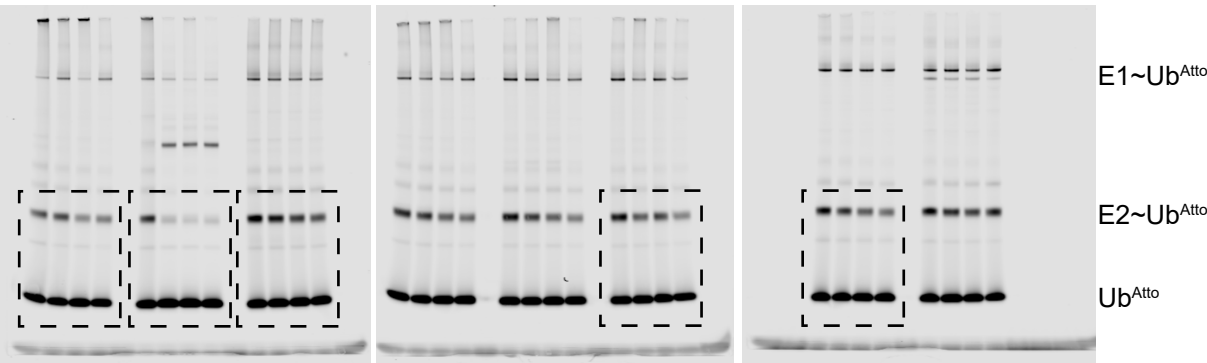

Supplement: Supplementary file 4 [file LSA-2019-00295_SdataFS2.pdf]

Figure 6 Source data

6A

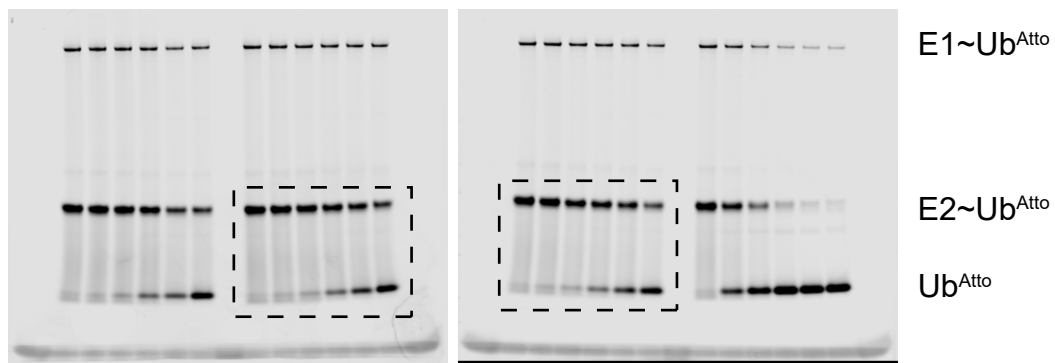

6C

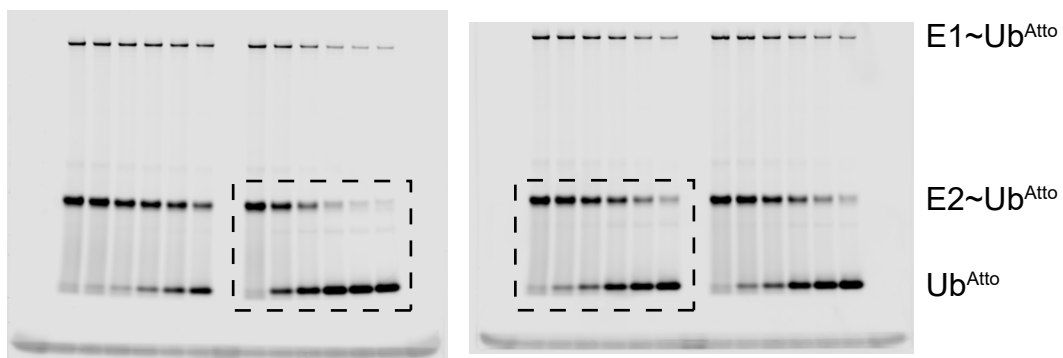

6E

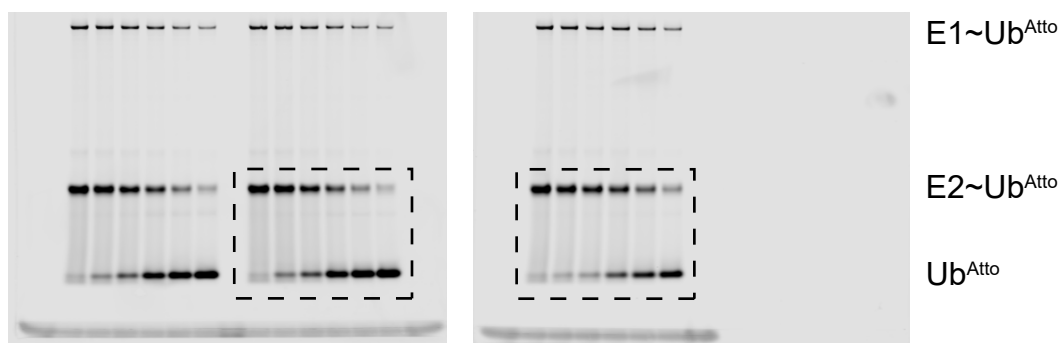

Supplement: Supplementary file 5 [file LSA-2019-00295_SdataF6.pdf]
