# Supplementary material for: Characterisation of class VI TRIM RING domains: linking RING activity to C-terminal domain identity
Source: Life Sci Alliance. 2019 Apr 26;2(3):e201900295. doi: 10.26508/lsa.201900295 (PMC6487577; doi:10.26508/lsa.201900295)
Supplement: Supplementary file 2 [file LSA-2019-00295_SdataFS1.pdf]

Figure S1 source data

S1A

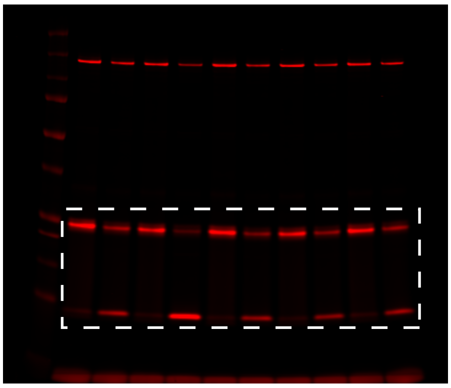

S1C

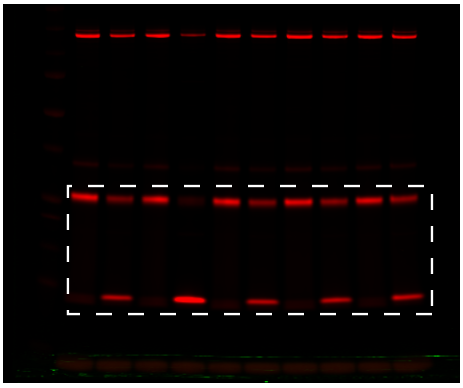

E1~Ub^{Atto}

E2~Ub^{Atto}

Ub^{Atto}

S1E

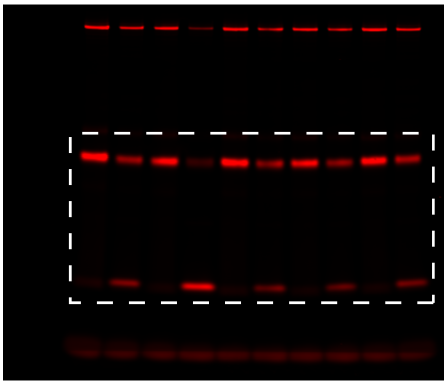

E1~Ub^{Atto}

E2~Ub^{Atto}

Ub^{Atto}
